# Toward Precision Medicine: How Far Is the Goal?

**DOI:** 10.3390/ijms17020245

**Published:** 2016-02-17

**Authors:** Gloria Ravegnini, Sabrina Angelini

**Affiliations:** Department of Pharmacy and Biotechnology (FaBit), University of Bologna, via Irnerio 48, Bologna 40126, Italy; gloria.ravegnini2@unibo.it

The accomplishment of the Human Genome Project, followed by the availability of high-throughput technologies, has led to an impressive change in biomedical research. In particular, the emergence of new tools for genome analysis has contributed to our knowledge of the molecular mechanisms that might reasonably influence treatment and response. This knowledge has paved the way to the development of personalized medicine, or more correctly precision medicine, which aims to determine unique individuals’ molecular characteristics, allowing the selection of the best treatment, and reduction of adverse reactions. The concept of “personalized medicine” is strictly connected with *Pharmacogenetics*, a term coined in the 20th century by Friedrich Vogel; however, this topic is particularly relevant if we consider that in 2015 the US President, Barack Obama, launched “a new Precision Medicine Initiative” [1,2]. Despite personalized medicine through pharmacogenetics/pharmacogenomics analyses represents an attractive strategy in disease treatment and there is a great interest in the development of powerful approaches to be incorporated into clinical practice, persistent gaps do exist between published research and clinical application. The personalized medicine embraces different field of the medicine, from chronic-pathologic disorders, as cancer, to autoimmune diseases as lupus erythematosus or rheumatoid arthritis. The Special Issue “Pharmacogenetics and Personalized Medicine” [3] published in the *International Journal of Molecular Sciences* takes into account different topics focusing on genetic variability and drug toxicity and/or efficacy and genomic and miRNA profiling to predict prognosis and outcome. Specifically, the issue consists of sixteen manuscripts, ten of which are original research articles and six are reviews. Table 1 summarizes the papers part of the Special Issue.

The majority of the papers belonging to the special issue investigated the role of polymorphisms in treatment response. Indeed, the role of germline DNA variations in clinical outcome or in drug toxicity is undeniable. This is well reported by Ravegnini and collaborators that made a general portrait of imatinib response in gastrointestinal stromal tumor (GIST) describing both tumor and patient DNA contribution to the final outcome [6]. Similarly, Polillo *et al.* depicted the importance of polymorphisms in imatinib and other tyrosine kinase inhibitors response in chronic myeloid leukemia (CML) [4]. These studies highlighted that polymorphisms located in genes codifying for imatinib transporters, as ABCG2 or SLC22A, might be involved in the clinical response. Indeed, the active uptake of imatinib into GIST and CML cells is known to be mediated mainly by transporter proteins, as hOCT1, or OCTN, whereas the efflux is mediated by the ABC transporters, in particular ABCB1 or ABCG2 [20,21,22,23]. In this regard, some studies have highlighted the influence of genetic polymorphisms in transporter genes and imatinib efficacy. Similarly, allelic variations in transporter genes seem to be important in the methotrexate treatment in rheumatoid arthritis patients as reported by Lima *et al.* [15]. Another underestimated aspect of precision medicine is gathered by three independent authors, Ruiz, Franca and Zaza [8,9,10] that take into consideration the impact of therapy personalization in transplanted patients. In particular, while Franca and Zaza reviewed the state of the art in hematopoietic stem cell and in renal transplantations, respectively [9,10], Ruiz reported an analysis on the impact of genetic polymorphisms in lung transplanted patients receiving mycophenolic acid or tacrolimus [8].

In addition to the certain role of polymorphisms in treatment response, recently the involvement of epigenetic mechanisms in personalized medicine has been catching on. This theme is faced by Cacabelos and Duroux-Richard and their respective collaborators [11,14]. Cacabelos, in particular, presented the epigenetics mechanisms in the Alzheimer disease (AD) and aging dealing with the three major regulatory elements—DNA methylation, histone modifications and miRNA regulation—responsible for the control of metabolic pathways at the molecular level [11]. The study highlighted that pharmacoepigenetic studies should be incorporated in drug development and personalized treatments. Duroux–Richard focused the analysis on miRNA profiling evaluation with the aim to identify novel biomarkers in systemic lupus erythematosus (SLE), [14]. The study identified a specific miRNA signature and provided a deeper insight into SLE immune-pathogenesis. With respect to miRNA deregulation, the field is attracting a growing research interest. Indeed, their alteration is often correlated with the rise and development of many diseases, including cancer, both in solid, hematopoietic malignancies. In this view, Simeon *et al.* gave a little summary of this crucial aspect in their review explaining an initial approach toward precise medicine in primary plasma cell leukemia (PCL) [5]; the review describes the available literature concerning the genomic characterization and pharmacogenetics of plasma cell leukemia and discuss the genomic characteristics based on conventional approaches, such as karyotype and fluorescence *in situ* hybridization analyses, and new high-throughput technologies, such as SNP array, gene expression profiling, miRNA expression profiling, and whole exome sequencing [5].

Overall, the sixteen studies included in this Special Issue illustrate how many steps in different disciplines have been covered from the achievement of the Human Genome Project.

At the moment, very few genotype-driven dose-optimization studies have prospectively assessed response rate, efficacy and toxicity, and have been translated into clinical practice. Currently, the evidences are still too sparse to provide a solid relationship between germline variants and drug response, largely due to the small size population under evaluation and lack of validated predictive polymorphisms. Furthermore, the identification of pharmacogenetic markers, transferable to clinical practice, may be complicated by the inability to currently take into account the effects of somatic genome, tumor heterogeneity, epigenetic factors; the possible existence of additional unidentified predictive factors can further complicate the application of pharmacogenetics. For these reasons, there is still an ongoing need for precision medicine. In this context, with the advent of next-generation techniques many progresses have been made, but we are still far from the goal to create an individualized treatment according to his/her genotype. Only with a huge effort both economic and research working, it will be possible to dock this pivotal turning point. We therefore strongly believe that multi-centric and multi-disciplinary works, led by laboratory researchers and clinicians in close collaboration will permit to advance our understanding and knowledge in precision medicine. In conclusion, to answer the question of how far is the goal toward a personalized medicine, despite the extensive studies and some promising results, it is still unclear when and how pharmacogenetic testing will be routinely integrated into patient management.

## Figures and Tables

**Table 1:** Summary of the papers in the Special Issue.

References	Title	Main Topic	Type of Paper
Polillo *et al.* [4]	Pharmacogenetics of BCR/ABL Inhibitors in Chronic Myeloid Leukemia	Cancer	Review
Simeon *et al.* [5]	Molecular Classification and Pharmacogenetics of Primary Plasma Cell Leukemia: An Initial Approach toward Precision Medicine	Cancer	Review
Ravegnini *et al.* [6]	Personalized Medicine in Gastrointestinal Stromal Tumor (GIST): Clinical Implications of the Somatic and Germline DNA Analysis	Cancer	Review
Rama *et al.* [7]	Specific Colon Cancer Cell Cytotoxicity Induced by Bacteriophage E Gene Expression under Transcriptional Control of Carcinoembryonic Antigen Promoter	Cancer	Article
Ruiz *et al.* [8]	Impact of Single Nucleotide Polymorphisms (SNPs) on Immunosuppressive Therapy in Lung Transplantation	Transplantation	Article
Franca *et al.* [9]	Role of Pharmacogenetics in Hematopoietic Stem Cell Transplantation Outcome in Children	Transplantation	Review
Zaza *et al.* [10]	Personalization of the immunosuppressive treatment in renal transplant recipients: the great challenge in “omics“ medicine	Transplantation	Review
Cacabelos *et al.* [11]	Epigenetics of Aging and Alzheimer’s Disease: Implications for Pharmacogenomics and Drug Response.	Degenerative disease	Review
Parmeggian *et al.* [12]	Effect of Factor XIII-A G185T Polymorphism on Visual Prognosis after Photodynamic Therapy for Neovascular Macular Degeneration	Degenerative disease	Article
Conti *et al.* [13]	A polymorphism at the translation start site of the vitamin D receptor gene is associated with the response to anti-osteoporotic therapy in postmenopausal women from southern Italy	Degenerative disease	Article
Duroux-Richard *et al.* [14]	MicroRNA Profiling of B Cell Subsets from Systemic Lupus Erythematosus Patients Reveals Promising Novel Biomarkers	Autoimmune disease	Article
Lima *et al.* [15]	Pharmacogenomics of Methotrexate Membrane Transport Pathway: Can Clinical Response to Methotrexate in Rheumatoid Arthritis Be Predicted?	Autoimmune disease	Article
Li *et al.* [16]	PRRT2 Mutant Leads to Dysfunction of Glutamate Signaling	Inherited diseases	Article
Ciccacci *et al.* [17]	A pharmacogenetics study in Mozambican patients treated with nevirapine: full resequencing of TRAF3IP2 gene shows a novel association with SJS/TEN susceptibility	HIV	Article
Hajj *et al.* [18]	Genotyping test with clinical factors: better management of acute postoperative pain?	Post-operative pain	Article
He *et al.* [19]	PRRT2 mutations are related to febrile seizures in epileptic patients	Epilepsy	Article
